# Vanishing Bile Duct Syndrome in an Adult Patient: Case Report and Review of the Literature

**DOI:** 10.3390/jcm11123253

**Published:** 2022-06-07

**Authors:** Paolo Izzo, Gaetano Gallo, Massimo Codacci Pisanelli, Giuliano D’Onghia, Leonardo Macci, Raimondo Gabriele, Andrea Polistena, Luciano Izzo, Sara Izzo, Luigi Basso

**Affiliations:** 1 “Pietro Valdoni” Department of Surgery, Policlinico “Umberto I”, “Sapienza” University of Rome, Viale del Policlinico 155, 00161 Rome, Italy; p_izzo@hotmail.it (P.I.); massimo.codacci@uniroma1.it (M.C.P.); giuliano.donghia@gmail.com (G.D.); maccileonardo798@gmail.com (L.M.); raimondo.gabriele@uniroma1.it (R.G.); andrea.polistena@uniroma.it (A.P.); luciano.izzo@uniroma1.it (L.I.); luigi.basso@uniroma1.it (L.B.); 2 Department of Surgical Sciences, Policlinico “Umberto I”, “Sapienza” University of Rome, Viale del Policlinico 155, 00161 Rome, Italy; 3 Multidisciplinary Department of Medical-Surgical and Dental Specialties, Plastic Surgery Unit, Università degli Studi della Campania “Luigi Vanvitelli”, Piazza Luigi Miraglia 1, 80138 Napoli, Italy; sa_izzo@hotmail.it

**Keywords:** vanishing bile duct syndrome, cholestasis, ductopenia, ursodeoxycholic acid, prednisolone

## Abstract

Vanishing bile duct syndrome (VBDS) is a rare condition characterized by progressive loss, destruction, and disappearance of the intra-hepatic bile ducts, leading to cholestasis and ductopenia. The exact mechanism of development of VDBS has not been established yet. Diagnosis of VBDS mainly relies on clinical and disease related presentations, but liver biopsy is compulsory for diagnosis. Due to the low incidence reported in the literature, a standardized treatment of VDBS has not been established; hence, this rare condition must be managed at a tertiary liver referral center. Here, we report the management and treatment of VBDS of an 81-year-old woman without any history of exposure to antibiotics, neoplasms, etc.

## 1. Introduction

Vanishing bile duct syndrome (VBDS) is a rare condition characterized by progressive destruction and disappearance of the intrahepatic bile ducts in the portal area with resulting cholestasis [1]. Several causes have been associated with VBDS, including congenital and genetic diseases, autoimmune conditions, neoplasms, and infections. VBDS can be associated with common antibiotics including levofloxacin, meropenem, amoxicillin, and azithromycin [2]. Diagnosis of VBDS mainly relies on clinical and disease related presentations, but liver biopsy is compulsory for diagnosis. Here, we report a case of VBDS without any history of exposure to antibiotics, neoplasms, etc. Treatment with ursodeoxycholic acid and prednisolone has resulted in the improvement of clinical and biochemical cholestasis.

## 2. Case Presentation and Results

The present case report is developed according to the CARE checklist [3].

An 81-year-old woman presented to the department of Accident and Emergency Surgery of our hospital with fever and jaundice. Her past medical history included high blood pressure, on treatment with amlodipine. Social history was significant for tobacco use of 1–2 pack of cigarettes/week. This lady denied any intake of alcohol or of illicit drug(s), and reported neck pain, headache, and progressive worsening of her eyesight, on both sides of her visual fields, for the last five months. Two months before, she had acute bilateral impairment of her vision with bilateral papillary oedema treated by intravenous steroids in another hospital. At that time, anti-nuclear antibodies (ANA), extractable nuclear antigens (ENA), and antiphospholipid antibodies were all unremarkable. An MRI of the ophthalmic artery revealed occlusion of the central retinal arteries, and, at later follow ups, the papillary oedema had decreased but the eyesight had not improved. This lady also presented generalized weakness, but denied any chest pain, shortness of breath, orthopnea, headache, or abdominal discomfort, while her skin, albeit intact, presented to be jaundiced and dry. Distal arterial pulses were normal, and she had no patent clinical signs of hepatic encephalopathy. Liver function tests (LFTs) (Table 1) were remarkably altered and elevated, including aspartate aminotransferase ((AST) = 370 U/L (normal = 8–38)), alanine aminotransferase ((ALT) = 400 U/L (normal = 12–41)), gamma glutamyl transferase ((GGT) = 760 U/L (normal = 5–36)), and alkaline phosphatase ((ALP)= 1600 U/L (normal = 40–129)). Her blood tests also showed very high values of direct bilirubin (total bilirubin = 28.68 mg/dL (normal = 0.35–1), direct bilirubin = 25.57 mg/dL (normal = 0.15–0.35)). A chest X-ray did not reveal any abnormality of her lung parenchyma or infiltrates. Abdominal ultrasonography showed normal liver and pancreas, and not dilated intra and extrahepatic bile ducts. Serologic tests for A, B, and C viral hepatitis were all negative. IgM anti-EBV and cytomegalovirus tests were negative. An in-depth rheumatological evaluation was performed to look for a single cause of the ophthalmic alterations and hepatitis, but all tests were negative.

Parasitological and bile culture examination were also negative.

Since there was fever and leukocytosis, antibiotic therapy with piperacillin and tazobactam 2 g + 0.25 g/day was started for 5 days, without improvement in liver enzymes and bilirubin levels.

**Figure 1:**
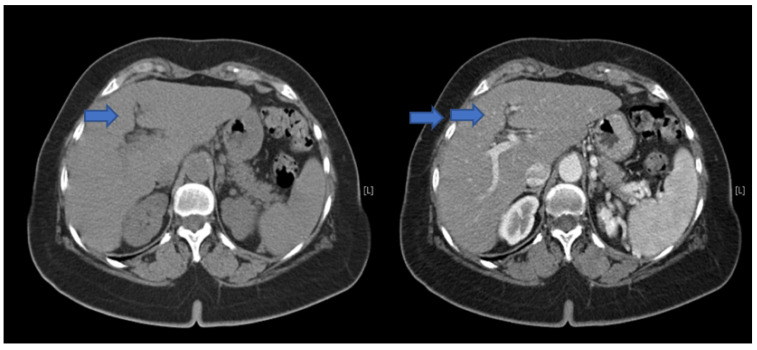
Combination or double image CT scan of the liver, without (blue arrow) and with (double blue arrow) contrast medium, which does not show significant alterations of the intra-hepatic vessels nor dilatations of the biliary ducts.

**Figure 2:**
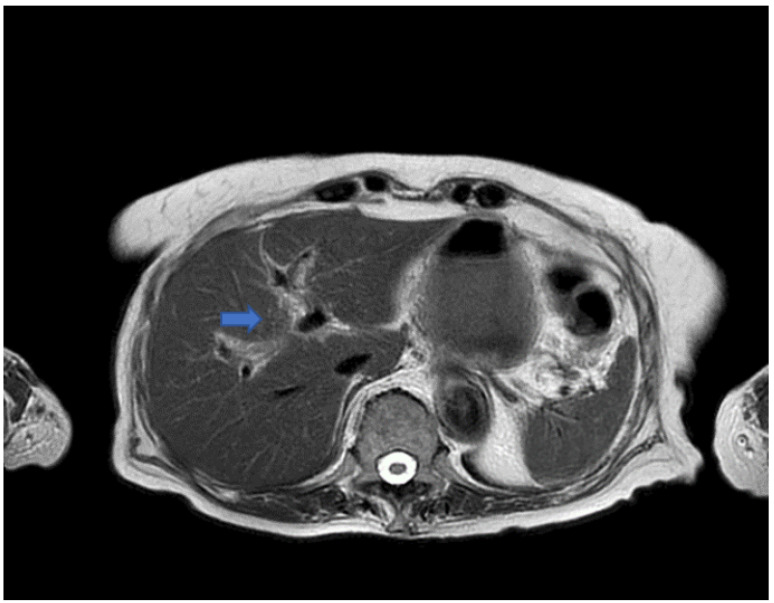
T2 weighted image (TR 2258 ms TE 100 ms) that does not show any alteration of caliber of the intrahepatic ducts (blue arrow).

**Figure 3:**
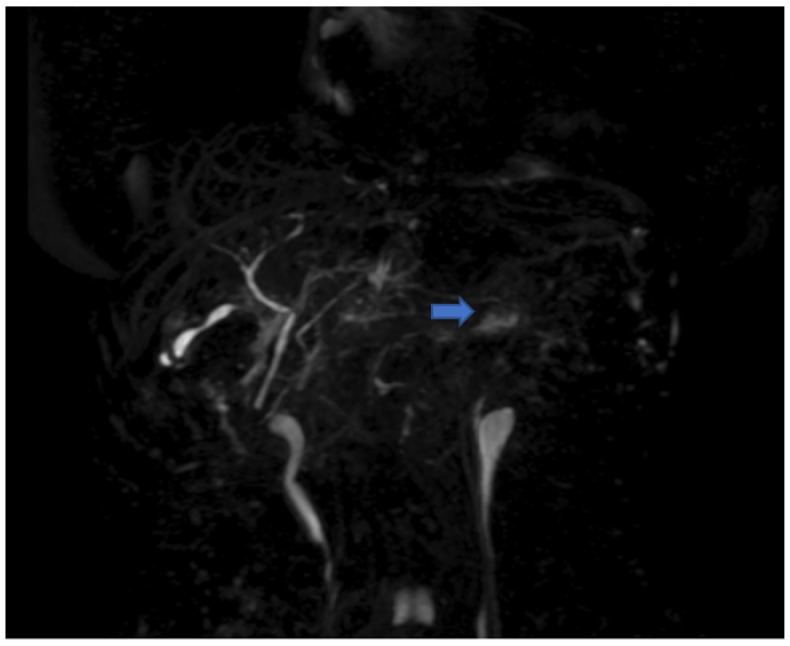
Magnetic resonance cholangiopancreatography showing poor uptake of the contrast medium, especially on the left lobe of the liver (blue arrow).

Since there were no abnormal findings of the abdominal CT scan (Figure 1), as well as of the MRI (Figure 2 and Figure 3), a punch biopsy of the liver was performed. Microscopic examination showed cholestasis and loss of the bile ducts, with more of 80% of small portal tracts abnormal and missing interlobular bile ducts. Most of small portal tracts showed evidence of periportal damage with fibrous expansion and marginal ductal proliferation.

Therapy was started with oral ursodeoxycholic acid at a dosage of 15 mg/kg/day (1125 mg) and oral prednisolone at a dosage of 1 mg/kg/day (75 mg), with consequent progressive reduction in the signs of liver inflammation (AST = 52, ALT = 90, GGT = 457, alkaline phosphatase = 284), while bilirubin values initially remained unchanged (Table 1).

Our patient was not in liver failure; however, conservative treatment was recommended by liver findings, with ursodeoxycholic acid (0.25 g 3 times a day) and prednisolone, gradually reduced to 15 mg/daily. Eventually, this patient was discharged home, and two weeks after her discharge, she showed a decrease in bilirubin to 5 mg/dL, and normal ALT and AST. This patient is regularly followed up, with LFTs (performed every three months) unremarkable after three years.

## 3. Discussion

VBDS is characterized by progressive loss, destruction, and disappearance of the intra-hepatic bile ducts, leading to cholestasis and ductopenia.

This condition was first described in 1988 in a series of three patients by Ludwig et al. as “idiopathic adulthood ductopenia” [4]. Ductopenia is defined as the absence of interlobular bile ducts in more than 50% of the small portal tracts in a liver biopsy specimen. The exact mechanism of development of VDBS has not been established yet. Various causes of VBDS have been identified and published, with different mechanisms of duct injury and loss. One of the most common causes of ductopenia is primary biliary cirrhosis (PBC), where the destruction of the epithelial cells of small bile ducts is T-cell-mediated, causing persistent cholestasis [5]. Intrahepatic cholestasis of pregnancy has rarely been reported, and mainly resolves spontaneously after childbirth [6], with no persistent increased bilirubin levels or ductopenia after delivery. More causes of VBDS with auto-immune mechanisms are acute and chronic hepatic rejection, sarcoidosis, and small duct primary sclerosing cholangitis [7]. Several medications may cause VBDS, such as penicillin, cephalosporins, sulfonamides, macrolides, and antifungal agents. Other authors have reported VBDS caused by antipsychotics, nonsteroidal anti-inflammatory drugs, omeprazole, and cardiovascular agents. In most of these instances, the patients fully recovered after discontinuing these drugs [8,9]. In our case, liver biopsy and histological examination played a key role in establishing VDBS, but it was not possible to recognize a specific cause of the disease. Due to the low incidence reported in the literature, a standardized treatment of VDBS has not been established; hence, this rare condition must be managed at a tertiary liver referral center [10,11]. In most cases, patients with VBDS respond to treatment of the underlying condition or to the simple withdrawal of the harming agent [8,9,12,13,14]. The use of steroids remains controversial, although these have been successfully used for severe cholestasis and VBDS [12,13,14], possibly for its potential beneficial effect in reducing the underlying inflammatory mechanisms. Other authors [8] consider corticosteroid ineffective. Ursodeoxycholic acid is the most frequently tested therapy [15], and it is thought to positively influence bile secretion and support the survival of cholangiocytes by inhibiting the intrinsic apoptosis pathway [16]. In the case we report, elevated total and direct bilirubin levels were found, without evidence of cholelithiasis or of biliary ductal dilatation. Liver biopsy plays a pivotal role in the diagnosis of VDBS. In the absence of other underlying conditions, VDBS has been treated with prednisone and ursodeoxycholic acid, and, in our experience, this therapy showed a prompt improvement of LFTs and a gradual decrease in the bilirubin levels.

## 4. Conclusions

Multiple factors can determine the development of VBDS, including common medications, autoimmune disorders, neoplasms, and infections, but, in most cases, it is impossible to identify the specific etiology of VBDS.

Biopsy and microscopic examination are crucial in establishing the diagnosis, but no standard therapeutic guidelines for the treatment of VBDS have, so far, been issued.

Ursodeoxycholic acid has been demonstrated to improve clinical and biochemical cholestasis in most instances and, in our case, the addition of prednisolone reduced the alterations of LFTs, and slowly reduced bilirubin levels.

## Figures and Tables

**Table 1:** Trend of LFTs during admission.

Day after Admission	AST	ALT	gammaGT	Alkaline Phosphatase	Total Bilirubine	Direct Bilirubine
1st	370 U/L	400 U/L	760 U/L	1600 U/L	28.68 mg/dL	25.57 mg/dL
7th *	320 U/L	380 U/L	771 U/L	1523 U/L	32.15 mg/dL	30.12 mg/dL
10th	152 U/L	170 U/L	579 U/L	780 U/L	30.10 mg/dL	29.45 mg/dL
14th	52 U/L	90 U/L	457 U/L	284 U/L	29.65 mg/dL	27.78 mg/dL
18th	35 U/L	58 U/L	123 U/L	201 U/L	15.12 mg/dL	13.82 mg/dL
23rd	21 U/L	23 U/L	78 U/L	104 U/L	7.55 mg/dL	5.12 mg/dL
25th	17 U/L	19 U/L	56 U/L	98 U/L	5.00 mg/dL	3.78 mg/dL

* Prednisone and ursodeoxycholic acid were started on 7th day from admission.
